# Structural basis for substrate specificity of methylsuccinyl-CoA dehydrogenase, an unusual member of the acyl-CoA dehydrogenase family

**DOI:** 10.1074/jbc.RA117.000764

**Published:** 2017-12-22

**Authors:** Thomas Schwander, Richard McLean, Jan Zarzycki, Tobias J. Erb

**Affiliations:** From the ‡ Department of Biochemistry and Synthetic Metabolism, Max-Planck-Institute for Terrestrial Microbiology Marburg, Karl-von-Frisch-Strasse 10, D-35043 Marburg, Germany and; the § LOEWE Center for Synthetic Microbiology (SYNMIKRO), D-35043 Marburg, Germany

**Keywords:** enzyme purification, enzyme structure, enzyme mutation, enzyme kinetics, enzyme catalysis, X-ray crystallography, bioengineering, flavin adenine dinucleotide (FAD), acyl-CoA dehydrogenase, ethylmalonyl-CoA pathway, glutaryl-CoA dehydrogenase, isobutyryl-CoA dehydrogenase, mesaconyl-CoA

## Abstract

(2*S*)-methylsuccinyl-CoA dehydrogenase (MCD) belongs to the family of FAD-dependent acyl-CoA dehydrogenase (ACD) and is a key enzyme of the ethylmalonyl-CoA pathway for acetate assimilation. It catalyzes the oxidation of (2*S*)-methylsuccinyl-CoA to α,β-unsaturated mesaconyl-CoA and shows only about 0.5% activity with succinyl-CoA. Here we report the crystal structure of MCD at a resolution of 1.37 Å. The enzyme forms a homodimer of two 60-kDa subunits. Compared with other ACDs, MCD contains an ∼170-residue-long N-terminal extension that structurally mimics a dimer–dimer interface of these enzymes that are canonically organized as tetramers. MCD catalyzes the unprecedented oxidation of an α-methyl branched dicarboxylic acid CoA thioester. Substrate specificity is achieved by a cluster of three arginines that accommodates the terminal carboxyl group and a dedicated cavity that facilitates binding of the C2 methyl branch. MCD apparently evolved toward preventing the nonspecific oxidation of succinyl-CoA, which is a close structural homolog of (2*S*)-methylsuccinyl-CoA and an essential intermediate in central carbon metabolism. For different metabolic engineering and biotechnological applications, however, an enzyme that can oxidize succinyl-CoA to fumaryl-CoA is sought after. Based on the MCD structure, we were able to shift substrate specificity of MCD toward succinyl-CoA through active-site mutagenesis.

## Introduction

The methylsuccinyl-CoA dehydrogenase (MCD)^3^ is a member of the flavin-dependent acyl-CoA dehydrogenase (ACD) family. MCD catalyzes the oxidation of the α-branched (2*S*)-methylsuccinyl-CoA into the corresponding *trans*-2-enoyl-CoA thioester, mesaconyl-CoA. The enzyme plays a crucial role in the ethylmalonyl-CoA pathway for acetyl-CoA assimilation in many α-proteobacteria (*e.g. Rhodobacter sphaeroides*, *Methylobacterium extorquens*, and *Paracoccus denitrificans*), actinobacteria (*e.g. Streptomyces* spp. and *Frankia* spp.), and possibly spirochaetes (*e.g. Leptospira* spp.) (1).

Other described members of the ACD family are short-chain acyl-CoA dehydrogenase (SCAD), medium-chain acyl-CoA dehydrogenase (MCAD), long-chain acyl-CoA dehydrogenase (LCAD), and very long-chain acyl-CoA dehydrogenase (VLCAD), which are involved in the β-oxidation of fatty acids. Additionally, isovaleryl-CoA dehydrogenase (IVD), isobutyryl-CoA dehydrogenase (IBD), branched short chain acyl-CoA dehydrogenase (BSCAD), and glutaryl-CoA dehydrogenase (GDH) are involved in the degradation of amino acids like leucine, valine, isoleucine, tryptophan, and lysine (2–5). A feature common to all of these enzymes is the presence of one non-covalently bound FAD cofactor per active site.

The α,β-desaturation of the CoA thioester is initiated by a proton abstraction from the α-carbon by a conserved catalytically active glutamate and a hydride transfer from the β-carbon to the N5 of the FAD cofactor (6, 7). The reduced co-factor is reoxidized by two sequential one-electron transfers to electron transfer flavoproteins (ETFs), which in turn deliver the electrons to the membrane-bound electron transport chain for energy conservation (8).

For many members of the ACD family, structures were solved by X-ray crystallography, and almost all of them form homotetramers (dimers of dimers) consisting of four ∼43-kDa subunits (9). An exception is the VLCAD, which forms a dimer of two ∼67-kDa subunits (10). All enzymes in the ACD family share an overall homologous central fold, composed of an N-terminal α-helix domain, an intermediate β-barrel domain, and a C-terminal α-helix domain. Again, the VLCAD represents an exception, as it comprises the central fold of ACDs but also possesses a C-terminal ∼180-amino acid extension forming an additional α-helix domain, responsible for dimer stability and the anchoring of the protein to the membrane (10).

The related family of acyl-CoA oxidases (ACOs) are responsible for the β-oxidation of fatty acids mainly in the peroxisomes of eukaryotes. ACOs also contain FAD as cofactor and belong to the same superfamily of enzymes as ACDs. Instead of transferring the electrons from the oxidation reaction to ETFs, however, ACOs use molecular oxygen as a direct electron acceptor. Similar to VLCAD, these oxidases form homodimers that possess C-terminal extensions. Nevertheless, ACOs lack the transmembrane helix anchor and are thus soluble and not membrane-bound. The catalytic domains of ACOs resemble to some extent the central fold of ACDs (11).

The different members of the ACD family exhibit substrate specificities toward distinct groups of CoA thioesters, respectively, which are conveyed by defined active-site architectures. The accommodation of all of these different substrates within the same fold is therefore of particular interest. In SCAD (PDB code 1JQI), the active site is restricted by an isoleucine residue from helix G (12), whereas MCAD (PDB code 3MDE) possesses prolines in the same α-helix. This results in a change of the direction of the helix in MCAD, which widens the substrate cavity to accommodate acyl-CoA thioesters of medium chain length (4–16 carbons) (13, 14). Bulkier residues at the active site of MCADs are substituted by glycines in VLCAD (PDB code 3B96), which opens the substrate pocket beyond these residues to allow the accommodation of acyl-CoA thioesters with up to 24 carbons in VLCAD (10). In the case of IVD (PDB code 1IVH), the catalytic glutamate residue is substituted by an alanine. A glutamate from the opposite α-helix G assumes the position and function of the canonical glutamate. This results in a lateral expansion of the substrate pocket and enables the accommodation of the β-branched substrate (15). Similarly, the active-site cavity in IBD (PDB code 1RX0) is narrower near the top and wider at the base to accept the α-branched substrate (16). In GDH (PDB code 3MPI), an arginine is important for binding of the C5 carboxylic acid moiety of the CoA thioester substrate (17, 18).

Compared with all other acyl-CoA esters discussed above, (2*S*)-methylsuccinyl-CoA represents a substrate with mixed chemical properties. It is short (C4) but possesses a terminal carboxylic acid group as well as an additional C2-methyl branch. To rationalize the function of MCD, it is important to decipher how substrate specificity is conferred in MCD and how a smaller substrate like succinyl-CoA, which lacks the methyl branch, is excluded from the active site.

**Figure 1.**
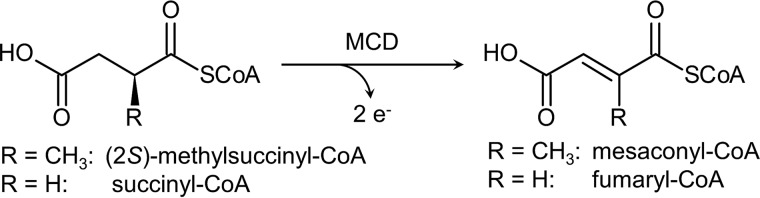
**Reaction catalyzed by MCD.** The natural substrate of MCD is the branched (2*S*)-methylsuccinyl-CoA, which is converted to mesaconyl-CoA. MCD also accepts the unbranched succinyl-CoA, but with a ∼200-fold lower catalytic rate.

Here we report the high resolution structure of MCD from *P. denitrificans* (PdMCD) that catalyzes the unprecedented α,β-desaturation of an α-methyl branched dicarboxylic acid CoA thioester. We used the structural information to rationalize the substrate specificity of MCD. This allowed us to engineer a MCD variant that exhibits improved catalytic efficiency with succinyl-CoA and a decreased efficiency with the original substrate (2*S*)-methylsuccinyl-CoA (Fig. 1). In a recent study, an oxidase function was introduced into the MCD scaffold. The high-resolution structure of MCD now provides a more detailed explanation for the engineered reactivity with oxygen (19).

## Results

### The core domain of PdMCD adopts the canonical fold of ACDs

PdMCD forms a homodimer, consistent with our gel filtration analysis (Fig. S1). The protein crystallized in the space group P 2_1_ 2_1_ 2_1_ with one dimer per asymmetric unit. The obtained well-ordered high-resolution electron density map allowed us to model almost the entire polypeptide chain (missing only 10 N-terminal residues) as well as the FAD cofactor in both active sites (Table 1).

**Table 1:** **Data collection and refinement statistics for PdMCD crystal structure**

**PDB code**	6ES9
Ligands	Flavin adenine dinucleotide, coenzyme A, sulfate

**Data collection**	
Wavelength	0.979
Space group	*P* 2_1_ 2_1_ 2_1_
Cell dimensions	
*a*, *b*, *c* (Å)	81.60, 105.19, 138.94
α, β, γ (degrees)	90.00, 90.00, 90.00
Resolution range (Å)	17.57–1.37 (1.42–1.37)
No. of observations	
Total	499,135 (49,543)
Unique	249,876 (24,803)
Redundancy	2.0 (2.0)
Mean *I*/σ*(I)*	15.25 (1.47)
*CC*½ (%)	99.9 (61.6)
Completeness (%)	99.92 (99.97)

**Refinement**	
*R*_work_/*R*_free_	0.1760 / 0.1847
No. of atoms	
Protein	8375
Ligands	180
Solvent	1389
Mean *B*-factors	
Protein	20.52
Ligands	24.86
Solvent	31.31
Root mean square deviations	
Bond lengths (Å)	0.016
Bond angles (degrees)	1.41
Ramachandran	
Favored (%)	98.53
Allowed (%)	1.29
Outliers (%)	0.18

*^a^* Numbers in parentheses indicate values for highest-resolution shell.

The enzyme crystallized only in the presence of mesaconyl-CoA, the reaction product of MCDs. Although we could observe some additional electron densities in the active sites, they were too ambiguous to model any ligand with confidence. Nevertheless, we also observed electron densities at the surfaces of each monomer that allowed the modeling of the adenosyl phosphate parts of CoA or its derivative. The adenine ring is taking part in cation-π stacking interactions between Arg-81 and Trp-476. This interaction might stabilize helix H, which is involved in a crystal contact. This may explain why crystals only formed in the presence of mesaconyl-CoA.

**Figure 2.**
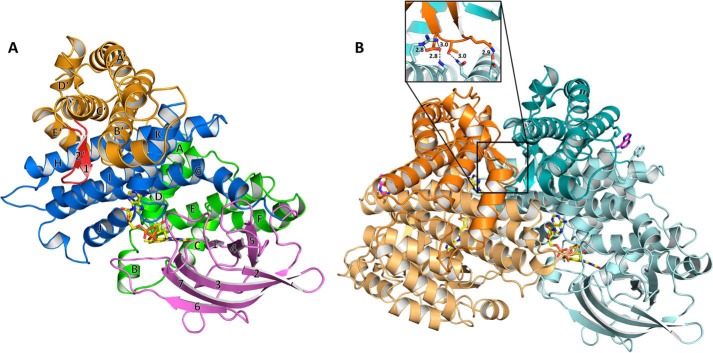
**Overall structure of PdMCD.** *A*, one monomeric subunit of PdMCD consists of five domains. The central fold found in other ACDs consists of an initial α-helical domain (*green*), an intermediary β-barrel domain (*pink*), and a C-terminal α-helical domain (*blue*). The N-terminal extension of PdMCD is shown in *orange* with the unique β-hairpin motif in *red*. The labeling of the secondary structure elements is in accordance with other ACDs. *B*, PdMCD forms a homodimer, and each subunit contains one FAD (*yellow*). The subunits are shown in *orange* and *cyan* with the N-terminal extension in *darker shades*, respectively. Adenosyl phosphate moieties were found to be bound on the surface of each subunit between Arg-81 and Trp-476. This interaction might stabilize helix H, which is involved in a crystal contact. The dimeric form of PdMCD is strengthened by the β-hairpin motif in the N-terminal extension (*highlighted* in the *box*), which forms hydrogen bonds with the opposite subunit.

**Figure 3.**
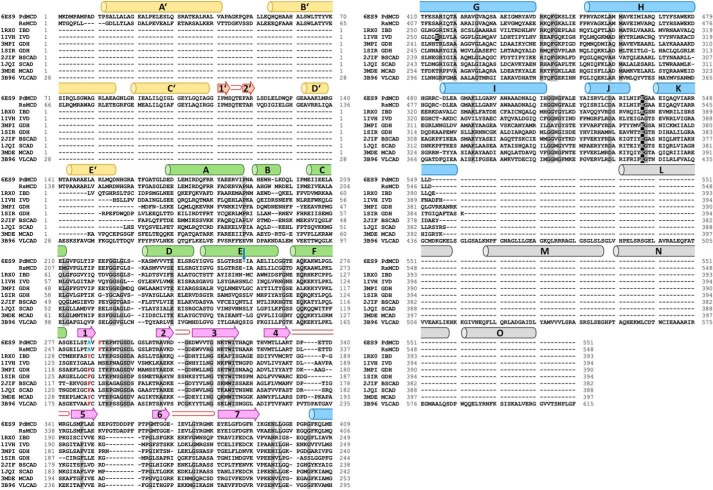
**Sequence alignment of PdMCD, RsMCD, and ACDs with known structures.** The sequences were aligned, and the secondary structure elements are indicated (α-helices with *barrels*, β-sheets with *arrows*, and turns in *red*). The labeling of the secondary structure element follows the convention for canonical ACDs. The N-terminal domain of MCD is shown in *orange*. The central fold of all known ACDs is divided into two α-helical domains (*green* and *blue*) and an intermediary β-barrel domain (*cyan*). The secondary structure elements of the C-terminal extension of VLCAD is indicated in *gray*. Identical residues are *shaded* in *gray*, and the active-site glutamate is *highlighted* in *black*. The α-helix E shows a single amino acid deletion like in IVD and MCAD (indicated by a *blue arrow*). The Phe-287 in PdMCD and Phe-284 in RsMCD are shifted by two residues toward the C terminus, and the otherwise conserved phenylalanine/tyrosine at this position is replaced with an alanine (indicated in *red* and *blue letters*). *BSCAD*, short/branched chain acyl-CoA dehydrogenase.

The core domain of PdMCD consists of ∼390 amino acids and assumes the canonical central fold known from other tetrameric ACDs. Therefore, we followed the conventional secondary structure labeling of ACDs for the α-helical domain (helices A–F), the intermediary β-barrel domain (strands 1–7), and the C-terminal α-helical domain (helices G–K). The only difference to the central fold of other ACDs is a ∼10-residue-long loop region connecting β-strands 5 and 6. This small loop is flanked by prolines (one N-terminal and three C-terminal) and contains a motif of three aspartates in a row (Figs. 2 and 3).

### An N-terminal extension in PdMCD mimics the canonical dimer–dimer interface of ACDs

**Figure 4.**
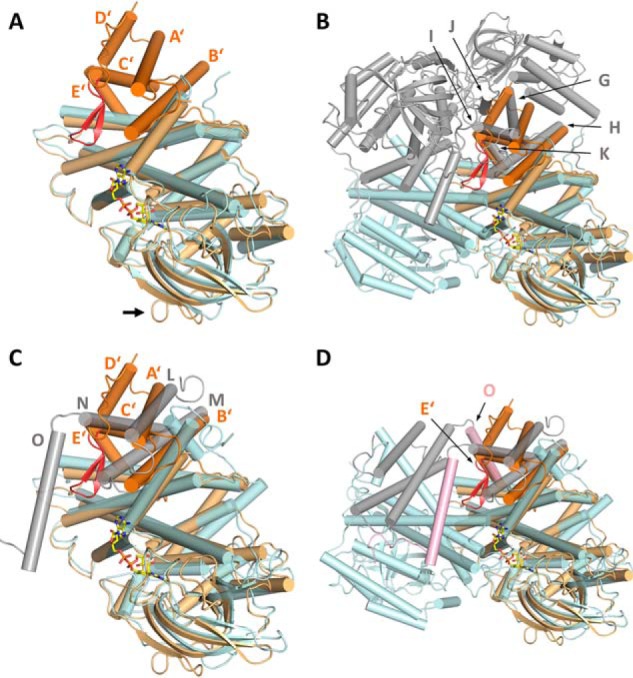
**Superposition of PdMCD with other ACDs.** *A*, structural alignment of a PdMCD monomer in orange (N-terminal domain in *dark orange*) with the monomer of MCAD in *cyan* (PDB code 3MDE) (1.55 Å over 350 Cα atoms per MCD monomer). The central fold of ACDs is conserved in the C-terminal domain of PdMCD. An extended loop region between β-strands 5 and 6 is indicated with an *arrow. B*, superposition of the PdMCD monomer with the tetramer (dimer of dimers in *cyan* and *gray*, respectively) of MCAD. The N-terminal extension of PdMCD aligns with the opposite monomer of MCAD and complements the dimeric interface. The unique β-hairpin motif (*red*) is located in a cavity at the interface, which can be found in all other ACDs known so far. *C*, superposition of a PdMCD monomer in *orange* (N-terminal domain in *dark orange*) with a monomer of VLCAD in *cyan* (PDB code 3B96) (1.85 Å over 313 Cα atoms). VLCADs have a C-terminal extension (*gray*), which aligns with the N-terminal extension from PdMCD and also complements the dimeric interface. *D*, overlay of a PdMCD monomer with a VLCAD dimer (N-terminal domain in *cyan*; C-terminal domain in *gray*). The subunits of VLCAD dimers also interact via α-helix O (*pink*). This feature is lacking in PdMCD, but the neighboring subunits additionally interact via the β-hairpin motif (*red*).

In contrast to canonical tetrameric ACDs, PdMCD possesses an additional ∼170-amino acid extension at the N terminus. We superposed the monomer of PdMCD with a monomer of MCAD (PDB code 3MDE). Whereas the C terminus of PdMCD (∼390 residues) aligned well with monomeric MCAD, the N terminus is organized in an additional domain. This domain comprises helices A′–C′, a small intermediate β-hairpin motif (strands 1′ and 2′), followed by helices D′–E′ (Fig. 4*A*).

However, when superimposing the PdMCD monomer with the MCAD tetramer, it becomes apparent that the N-terminal ∼170-residue extension of PdMCD structurally mimics the dimer–dimer interface of MCAD. The α-helices A′–E′ of PdMCD align well with the α-helices G–K from a neighboring MCAD subunit (Fig. 4*B*). Exceptions are a small β-hairpin motif (not found in MCAD) and α-helix D′ of PdMCD, which only loosely aligns with α-helix J from MCAD.

By emulating the dimer–dimer interface found in the tetrameric ACDs, the N-terminal extension of PdMCD probably stabilizes the PdMCD dimer. The PdMCD dimer is further stabilized by additional contacts provided by the unique intermediate β-hairpin motif of the N-terminal extension, In PdMCD, the β-hairpin features hydrophilic residues that can form hydrogen bonds and salt bridges to the central fold of the opposite subunit, whereas ACDs usually exhibit a small cavity at the corresponding position (Figs. 2*B* and 4). Indeed, replacing Glu-117 of the β-hairpin with an alanine leads to a destabilization of the PdMCD dimer, as shown by analytical size-exclusion chromatography (Fig. S1).

All residues from the N-terminal extension of PdMCD (helices A′–E′) show ∼20% sequence identity (32.5% sequence similarity) to the structurally homologous residues from the central fold (helices G–K), indicating that the N-terminal extension originated from a partial gene duplication. The same is assumed for the C-terminal extension found in VLCAD that also mimics a dimer–dimer interface (10). Superposition of PdMCD with the structure of VLCAD (PDB code 3B96) demonstrates that α-helices A′–C′ of PdMCD align well with α-helices L–N of VLCAD, whereas α-helix D′ of PdMCD does not have a counterpart in VLCAD, and α-helix E′ from PdMCD lines up with the α-helix O from another subunit of the VLCAD dimer (Fig. 4). Similar to VLCAD, PdMCD also superimposes with dimeric ACOs that possess a comparable C-terminal extension (Fig. S2). Interestingly, a similar mimicry of a dimer interface was also recently found in the aldehyde dehydrogenase superfamily (20) and might be a more common structural feature in enzyme structures.

### The active site

**Figure 5.**
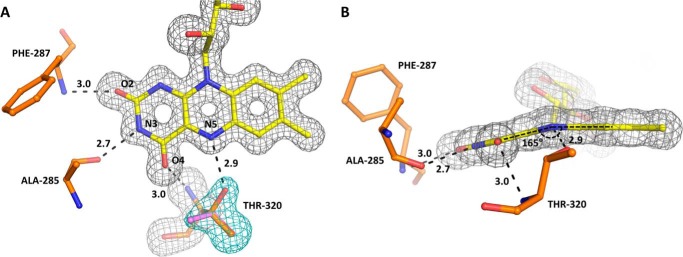
**Superposition of *F_o_* − *F_c_* electron density simulated annealing omit maps on a refined FAD and Thr-320.** *A*, omit map at 3.0 σ for the FAD cofactor and Thr-320 of PdMCD. Thr-320 forms a hydrogen bond to the N5 of the FAD. The electron density around this residue (*cyan*) indicates that the threonine can assume a different rotamer conformation (*purple*) where the hydrogen bond is broken. The FAD cofactor forms additional hydrogen bonds with the polypeptide backbone. *B*, the isolalloxazine ring of FAD assumes a distinct “butterfly-like” conformation (angle of 165°), which can be observed in other ACDs as well. This conformation is assumed to indicate a shift in the electron potential of the cofactor.

**Figure 6.**
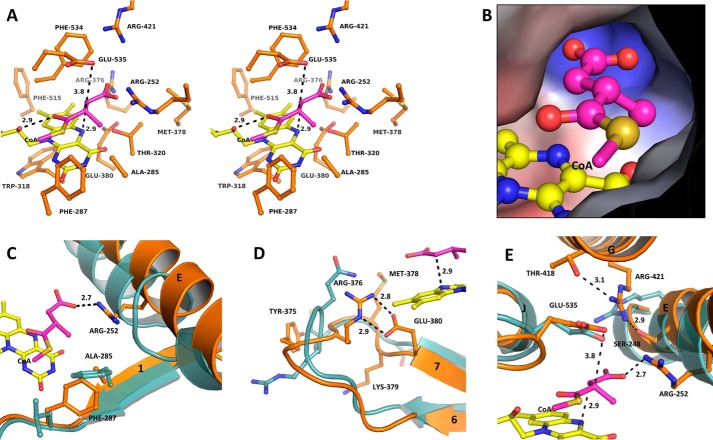
**Active-site architecture of PdMCD.** *A*, *wall-eyed stereo view* of the active-site residues. The acyl moiety of (2*S*)-methylsuccinyl-CoA was fitted into the active site according to the conserved positioning of substrates found in other ACDs. The carbonyl group of the thioester bond forms a hydrogen bond to the 2′-hydroxyl of the FAD ribityl chain and the backbone amine of the catalytic Glu-535. The C2 of the substrate is positioned below Glu-535 for proton abstraction, and the C3 is positioned above the N5 of FAD for the hydride transfer. The base of the active-site cavity is lined by Arg-252, Arg-376, and Arg-421 for binding of the carboxylic acid group of the substrate. *B*, the interior surface of the active site shows a distinct pocket for the accommodation of the C2-methyl group, and the base of the cavity is positively charged for the binding of the carboxylic acid of the substrate. *C*, overlay of PdMCD (*orange*) with VLCAD (PDB code 3B96) (*cyan*). The α-helix E of PdMCD is shifted away from the active site and a single residue deletion (as can be found in IVD and MCAD) prevents a perturbation of this helix. A conserved phenylalanine/tyrosine in other ACDs is shifted in PdMCD. This shift allows the formation of the cavity to accommodate the C2-methyl branch of the substrate by Ala-285. Moreover, Phe-287 partially assumes an analogous position to the conserved phenylalanine/tyrosine in other ACDs. *D*, overlay of PdMCD (*orange*) with MCAD (PDB code 3MDE) (*cyan*), indicating a repositioned loop between β-strands 6 and 7. This leads to the formation of a salt bridge between Glu-380 and Arg-376, which is thereby positioned within the active site. *E*, overlay of PdMCD (*orange*) with GDH (PDB code 3MPI) (*cyan*). The conserved catalytic glutamate is found in all ACDs (except IVD). In GDH, a salt bridge to the substrate glutaryl-CoA is formed with an arginine from α-helix E. In the case of PdMCD, Arg-421 is in an analogous position but originates from α-helix G. Arg-421 is held in place by Ser-248 and Thr-418. The Arg-252 from α-helix E is within interaction distance of the fitted methylsuccinyl-CoA and may assume a different conformation upon substrate binding.

The cofactor FAD binds to PdMCD in a “drawn-out” conformation, as observed for other ACDs (10, 13, 16, 18). Both subunits of the MCD dimer contribute to the binding of FAD, which adopts a “butterfly-like” conformation in respect to the isoalloxazine ring. This conformation for FAD was observed in other ACDs and is suggested to influence the redox potential of FAD for catalysis (21). The bending of the isoalloxazine ring is probably caused by hydrogen bonding to the polypeptide backbone (O_2_ with amine of Phe-287, O4 with amine of Thr-320, and N3 with carbonyl of Ala-285) (Fig. 5) and stereoelectronic interaction with aromatic residues (Phe-287, Trp-318, Phe-515, and Phe-534) (Fig. 6*A*).

Interestingly, in PdMCD, Phe-287 is shifted two residues toward the C terminus compared with the canonical phenylalanine/tyrosine in other ACDs (VLCAD, Phe-174; MCAD, Tyr-132; SCAD, Phe-128; and IBD, Tyr-136) (Fig. 3). However, the aromatic ring of Phe-287 in PdMCD still partially assumes the same space as the canonical aromatic rings of the other ACDs. Replacing the conserved aromatic residue by an alanine (Ala-285) and shifting the phenyl residue probably provides more space at the substrate-binding pocket in PdMCD (discussed below) (Fig. 6*C*). The situation resembles the one in IBD (PDB code 1RX0). There, another conserved aromatic residue found in ACDs (PdMCD, Phe-534; VLCAD, Phe-421; MCAD, Tyr-375; and SCAD, Tyr-367) is replaced with an isoleucine (Ile-375 in IBD), which widens the cavity for substrate accommodation (16). Trp-216 of IBD is oriented to partially assume the position of the conserved tyrosyl/phenyl ring found in the other ACDs.

Another important residue for catalysis is a glutamate residue in position 535 in PdMCD that is conserved in other ACDs and was proposed to act as the catalytic base for the initial abstraction of a proton from the substrate. This conserved residue is positioned between α-helix J and K in canonical ACDs and in a homologous position in PdMCD (Fig. 6*E*). Only in IVD, this glutamate is relocated to α-helix G, but it still occupies the same space in relation to the substrate.

A significant difference in the active-site architecture of PdMCD is an amino acid deletion (Fig. 3) in α-helix E, which prevents a perturbation of helix E as in IVD, SCAD, VLCAD, and GDH. This situation is comparable with that in MCAD and IBD, resulting in a lateral widening of the active site to accommodate sterically more challenging substrates. In PdMCD, however, helix E is shifted >2 Å to the back of the active site compared with IBD and MCAD, providing even more space, probably to accommodate the terminal carboxylic acid group of methylsuccinyl-CoA (Fig. 6*C*).

The carboxylic acid group of the substrate could potentially interact with three arginine residues (Arg-252, Arg-376, and Arg-421) that are all in close proximity (Fig. 6*A*). Of those, Arg-421 can be compared with an invariant arginine in GDH (Arg-87) that forms a salt bridge to the carboxylic acid group of glutaryl-CoA and is essential for catalysis in human GDH (22). Arg-252 could take part in a similar interaction, which is supported by the fact that mutation of Arg-252 into alanine, glutamine, and notably also lysine resulted in inactive enzyme. Arg-376 finally is provided by the loop between β-strand 6 and 7 (from Leu-372 to Glu-380). This loop assumes a strikingly different conformation in PdMCD compared with other ACDs and is stabilized by various residue interactions (*i.e.* Tyr-375 with the backbone carbonyl of Gln-198, Lys-379 with Glu-279, and Tyr-243 with the backbone carbonyl of Tyr-375). As a result, Arg-376 is positioned within the active site, where it is fixed via a bidentate side-on interaction by Glu-380 (Fig. 6*D*).

### Substrate specificity

For ACDs the different substrate specificities achieved in the conserved fold are of particular interest. MCD is highly specific for (2*S*)-methylsuccinyl-CoA without detectable activity toward (2*R*)-methylsuccinyl-CoA, butyryl-CoA, and isobutyryl-CoA (1). Although the enzyme accepts succinyl-CoA as a substrate, the rate of catalysis is ∼200-fold lower compared with (2*S*)-methylsuccinyl-CoA, suggesting that the C2-methyl group is crucial for the correct positioning of the substrate.

Analogous to other ACDs, PdMCD shows a cleft on the surface that extends into the active site where the binding of the CoA thioester takes place. Although PdMCD was crystallized in the presence of the reaction product mesaconyl-CoA, only very weak electron density could be observed at the binding site, which did not allow the modeling of CoA or a derivative. However, it is well known from other ACDs that the thioester carbonyl oxygen forms hydrogen bonds with the 2′-hydroxyl group of the ribityl moiety of the riboflavin and the backbone amine hydrogen of the catalytic glutamate that acts as a base. Additionally, the C2 of the substrate is required to be positioned directly below this glutamate for efficient proton abstraction, whereas C3 of the substrate needs to be positioned above the N5 of the isoalloxazine ring of FAD for the hydride transfer (10, 13, 16, 18, 23).

Following these restrictions, we fitted (2*S*)-methylsuccinyl-CoA into the active site, assuming the terminal carboxyl group to be planar relative to the carbon backbone of the molecule and parallel to the isoalloxazine ring of FAD. This would also be the case for the oxidized product (mesaconyl-CoA), in which the double bond would stabilize the planar conformation by delocalization. The modeled substrate fitted the active site well, and its C2-methyl branch as well as the carboxylic acid group could be accommodated without any significant clashing (Fig. 6*B*).

### Structure-based active-site engineering

We investigated the role of the different residues for substrate specificity in the homologous MCD of *R. sphaeroides* (RsMCD), which we used previously for engineering efforts (19) and which proved to be a very stable and malleable homolog in our hands. RsMCD exhibits 88% amino acid sequence identity (95% similarity) in the central core to that of PdMCD, with nearly identical catalytic properties (Figs. S3 and S4).

**Figure 7.**
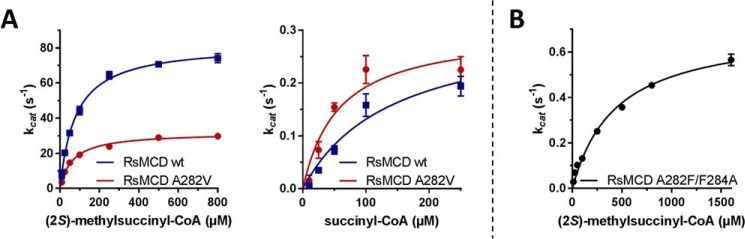
**Kinetic properties of RsMCD WT, A282V variant, and the A282F/F284A double variant (corresponding to Ala-285 and Phe-287 in PdMCD).** *A*, Michaelis–Menten kinetics of RsMCD WT and A282V variant with (2*S*)-methylsuccinyl-CoA and succinyl-CoA as substrate. The variant shows a decreased efficiency with (2*S*)-methylsuccinyl-CoA but has a slightly increased efficiency with succinyl-CoA. *B*, Michaelis–Menten kinetics of RsMCD A282F/F284A swapping mutant with (2*S*)-methylsuccinyl-CoA. This mutant only achieved 0.2% of relative catalytic efficiency with (2*S*)-methylsuccinyl-CoA and did not show measurable activity with succinyl-CoA. *Error bars*, S.D.

To understand substrate specificity of MCDs, we focused on the cavity formed by Ala-285 in PdMCD presumably accommodating the C2-methyl branch of (2*S*)-methylsuccinyl-CoA. Substitution of the equivalent Ala-282 in RsMCD by sterically more demanding residues like leucine, isoleucine, and phenylalanine yielded only inactive enzymes. These larger residues probably prevent positioning of the C2 branch but probably also negatively impact the interaction of the carboxyl group with Arg-249 (Arg-252 in PdMCD). In contrast, mutation of the alanine into valine still resulted in some residual activity with (2*S*)-methylsuccinyl-CoA. The catalytic efficiency of the A282V variant with (2*S*)-methylsuccinyl-CoA was decreased by 50% compared with the wildtype, which was mostly due to a decreased turnover number. On the other hand, the A282V variant exhibited an increased catalytic efficiency with unbranched succinyl-CoA due to a decrease in *K_m_* (Table 2 and Fig. 7*A*). This indicated that the formation of a productive Michaelis complex of the enzyme with branched (2*S*)-methylsuccinyl-CoA was impaired, whereas Michaelis complex formation with unbranched succinyl-CoA was enhanced. The A282V mutation did not influence the interaction of the enzyme with the artificial electron acceptor ferrocenium and did not show any oxidase activity (Figs. S5 and S6).

**Table 2:** **Michaelis–Menten kinetic parameters for the wildtype and mutant RsMCD with either (2*S*)-methylsuccinyl-CoA or succinyl-CoA as substrate** Ala-282 and Phe-284 correspond to Ala-285 and Phe-287 in PdMCD, respectively. ND, no detectable activity.

	(2*S*)-Methylsuccinyl-CoA			Succinyl-CoA		
	*k*_cat_	*K_m_*_(app)_	*k*_cat_/*K_m_*	*k*_cat_	*K_m_*_(app)_	*k*_cat_/*K_m_*
	*s*^−*1*^	μ*m*	*s*^−*1*^ *m*^−*1*^	*s*^−*1*^	μ*m*	*s*^−*1*^ *m*^−*1*^
RsMCD WT	82.3 ± 1.4	80 ± 5	1.0 × 10^6^ ± 0.1 × 10^6^	0.32 ± 0.06	141 ± 52	2.3 × 10^3^ ± 0.9 × 10^3^
RsMCD A282V	31.8 ± 0.6	64 ± 5	0.5 × 10^6^ ± 0.04 × 10^6^	0.30 ± 0.04	57 ± 20	5.3 × 10^3^ ± 2.0 × 10^3^
RsMCD A282L	ND	ND	ND	ND	ND	ND
RsMCD A282I	ND	ND	ND	ND	ND	ND
RsMCD A282F	ND	ND	ND	ND	ND	ND
RsMCD A282F/F284A	0.71 ± 0.03	439 ± 49	1.6 × 10^3^ ± 0.2 × 10^3^	ND	ND	ND
RsMCD A282F/F284V	ND	ND	ND	ND	ND	ND
RsMCD A282F/F284L	ND	ND	ND	ND	ND	ND

We also investigated the role of Phe-284 in RsMCD (Phe-287 in PdMCD), which is shifted toward the C terminus by two positions compared with other ACDs and is known to influence the redox potential of FAD (21). We substituted Phe-284 with an alanine, valine, or leucine within the A282F background of RsMCD, to reverse the C-terminal shift and mimic the canonical positioning of the residues as found in other ACDs. The double mutants did not show any activity with succinyl-CoA, with the exception of the A282F/F284A variant, which was still able to convert (2*S*)-methyl-succinyl-CoA, however with less than 0.2% of the relative catalytic efficiency (Table 2 and Fig. 7*B*). Presumably, the F284A mutation allows the Phe-282 residue introduced to adopt a rotamer conformation similar to that observed in other ACDs (Fig. 6*C*), so that only a small lateral cavity is formed, which is barely able to accommodate the C2-methyl group of the substrate.

## Discussion

The structure of PdMCD is unique among ACDs with an N-terminal extension to the conserved core and a specific activity toward the complex substrate (2*S*)-methylsuccinyl-CoA. The substrate's special demands are met by an active site that is able to accommodate the methyl group branching at the C2 as well as the carboxylic acid group of the substrate.

The inner surface at the base of the active-site cavity of PdMCD is positively charged through three arginines (Fig. 6*B*). A similar observation is made in the structure of GDH (PDB code 3MPI) that oxidizes glutaryl-CoA, which is structurally similar to (2*S*)-methylsuccinyl-CoA, slightly longer and unbranched. In GDH, Arg-87 (from α-helix E) provides a positive charge and forms a bidentate end-on interaction with the carboxyl group of glutaryl-CoA (18). In the case of PdMCD, Arg-421 is located in an analogous position but originates from α-helix G (Fig. 6*E*). In our model of the ternary complex, Arg-421 in PdMCD is at a distance of about 4 Å from the fitted carboxylic acid group of the substrate and could form a salt bridge. Another arginine, Arg-376, from the twisted loop between β-strands 6 and 7 constricts the lower base of the active site and could also contribute to substrate binding (Fig. 6*D*). Whereas Arg-376 and Arg-421 are fixed by either Glu-380 or Ser-248 and Thr-418, respectively, a third arginine at position 252 (from α-helix E) has no obvious interacting residue in the vicinity (Fig. 6*E*). Arg-252 apparently only forms a water bridge to Ser-283 and has a fairly close contact to the catalytic Glu-535. The latter, however, is a very unlikely interaction partner, as this would increase the p*K_a_* of the catalytic base. Mutagenesis showed that Arg-252 is essential for catalysis. It is therefore possible that Arg-252 undergoes a conformational change upon substrate binding, approaching the carboxylic acid group of (2*S*)-methylsuccinyl-CoA to form mono- or bidentate side-on interactions, similar to Arg-87 in GDH (18).

The active site is laterally expanded, forming a pocket, which could provide space for the C2-methyl group. This pocket is formed by Ala-285, Phe-287, and Arg-252 (Fig. 6*A*). A similar pocket is not present in other ACDs, where the Ala-285 is usually substituted by a conserved phenylalanine/tyrosine instead, which are known to tune the electropotential of FAD (21). Interestingly, an alternative phenylalanine (Phe-287) is found in PdMCD that appears shifted toward the C terminus and seems to partially assume a similar space as the conserved phenylalanine/tyrosine in the other ACDs (Fig. 6*C*). The C2-methyl group of the fitted substrate probably protrudes into the cavity opened up by Ala-285.

MCD apparently evolved toward preventing the oxidation of the unbranched structural homolog succinyl-CoA, which is an essential intermediate in central carbon metabolism. Preventing the nonspecific oxidation of succinyl-CoA is physiologically highly relevant, because this would otherwise deplete the succinyl-CoA pool in the oxidative tricarboxylic acid cycle and also lead to formation of the dead-end metabolite fumaryl-CoA. Thus, the enzyme needs to carefully distinguish between the presence and absence of a single methyl group and exclude the smaller substrate, which is not an easy task. The molecular basis for this ability probably arises from a lower binding energy for succinyl-CoA lacking the C2-methyl group. Therefore, the methyl group of (2*S*)-methylsuccinyl-CoA is probably crucial for the precise positioning of the α,β-carbon bond of the substrate during catalysis. The absence of the methyl group could render the succinyl-CoA more flexible and movable, which could affect correct positioning and lead to unproductive binding states. A similar observation was made for IBD, where propionyl-CoA is converted with only 5% of the relative catalytic rate compared with the true substrate isobutyryl-CoA. Here, the C2-methyl branch hinders the free rotation around the α-carbon in the active site and wedges the substrate into position for efficient catalysis (16).

Nevertheless, for different metabolic engineering (including the design of artificial pathways) and synthetic-biological applications, a soluble enzyme that catalyzes the α,β-carbon bond desaturation of succinyl-CoA to fumaryl-CoA is of interest. This reaction would allow a shortcut to the otherwise multienzyme reaction sequence via the citric acid cycle, which includes CoA ester hydrolysis, membrane-associated oxidation of succinate, and re-formation of a CoA ester. Partially closing the putative C2-binding pocket of MCD by replacing Ala-282 in RsMCD with valine allowed us to change substrate specificity toward succinyl-CoA, albeit at a reduced catalytic rate. This indicates that there is a potential to reverse the evolved substrate specificity of methylsuccinyl-CoA dehydrogenase.

We previously engineered RsMCD into an oxidase (RsMCO) through three amino acid substitutions. RsMCO was able to directly use molecular oxygen as an electron acceptor and catalyzed this reaction with ∼1.5% of relative activity compared with RsMCD WT (19). The structure of PdMCD allows us to rationalize the structural basis for the engineered oxidase activity in retrospect. A key mutation in engineering RsMCO was to replace Thr-317 (Thr-320 in PdMCD) with glycine, which is the canonical amino acid at this position in ACOs. The high resolution of the electron density map at the active site shows that Thr-320 in PdMCD can adopt different rotamer conformations, of which one forms a direct hydrogen bond to the N5 of the isoalloxazine ring of FAD (Fig. 5*A*). Because this residue is conserved in ACDs, it might not only exclude molecular oxygen from the active site but also influence the electropotential of FAD by stabilizing the semiquinone state of the cofactor. This could favor the two single-electron transfers to ETFs compared with the two-electron transfer to molecular oxygen that takes place in ACOs (24). Moreover, the engineered RsMCO also comprises a mutation of Trp-315 (Trp-318 in PdMCD) to phenylalanine, which probably increases solvent accessibility of the active site and also modulates the electropotential of FAD (21, 23). Additionally, Glu-377 (Glu-380 in PdMCD) was mutated to asparagine, which is not in direct contact with FAD but is conserved among ACOs and probably stabilizes the formation of the superoxide anion intermediate (25).

In conclusion, the crystal structure of PdMCD presented in this study provides new insights into the molecular basis of reaction and substrate specificity in the ACD superfamily and leads the way for engineering approaches to accommodate new electron acceptors and/or substrates at the active site of this highly abundant and relevant class of enzymes.

## Experimental procedures

### Materials

Chemicals were obtained from Sigma-Aldrich (Munich, Germany) and CARL ROTH GmbH (Karlsruhe, Germany). Biochemicals and materials for cloning and protein production were obtained from Thermo Fisher Scientific (St. Leon-Rot, Germany), New England Biolabs GmbH (Frankfurt am Main, Germany), and Macherey-Nagel GmbH (Düren, Germany). Primers were obtained from Eurofins MWG GmbH (Ebersberg, Germany). Materials and equipment for protein purification were obtained from GE Healthcare (Freiburg, Germany), Bio-Rad (Munich, Germany), and Merck Millipore GmbH (Schwalbach, Germany).

### Synthesis of CoA thioesters

Crotonyl-CoA and succinyl-CoA were synthesized from their respective anhydrides. Mesaconyl-CoA was synthesized via the mixed anhydride method, starting from the free acid and obtaining a mixture of the isomeric 2-methyl and 3-methyl branched thioester (26). Ethylmalonyl-CoA was produced with 15 units of crotonyl-CoA carboxylase from 6 mm crotonyl-CoA in 5 ml of reaction buffer containing 60 mm ammonium carbonate, pH 8, 50 mm NaHCO_3_, and 7 mm NADPH. All CoA thioesters were purified using an HPLC system (1260 Infinity, Agilent Technologies GmbH, Waldbronn, Germany) with a Gemini® 10-μm NX-C18 110-Å column (Phenomenex, Aschaffenburg, Germany) as described before (27). The concentration of CoA esters was quantified by determining the absorption at 260 nm (ϵ = 22.4 mm^−1^ cm^−1^ for unsaturated and ϵ = 16.4 mm^−1^ cm^−1^ for saturated CoA thioesters).

### Cloning and protein production

The gene coding for MCD from *P. denitrificans* was amplified using chromosomal DNA as a template. Two oligonucleotides introducing restriction sites (underlined) were designed upstream (5′-ACA TGC ATA TGA AGG ACA TGC CCG CGA TG-3′; NdeI) and downstream (5′-ATT ATG CGG CCG *C*CT AGT CCA GTA GCC TGC GTG C-3′; NotI) of the gene coding of MCD. PCR was performed with *Phusion*® high-fidelity DNA polymerase in GC-buffer for 35 cycles, including denaturation for 60 s at 98 °C, annealing for 30 s at 57 °C, and polymerization for 2 min at 72 °C. The PCR product was cloned into the pET28b vector for expression, resulting in plasmid pTE849. Competent *Escherichia coli* Rosetta pLys (DE3) were transformed with the plasmid, and 1-liter cultures were grown at 37 °C in TB medium with 50 μg of kanamycin ml^−1^ to an OD of ∼1, and expression was performed at 20 °C overnight after induction with 250 μm isopropyl 1-thio-β-d-galactopyranoside. The cells were harvested by centrifugation at 5000 rcf for 10 min and resuspended in 2 times the volume of lysis buffer (20 mm Tris-HCl, pH 7.9, 500 mm NaCl, 10% glycerol) and eventually stored at −20 °C.

The cloning of the *mcd* gene from *R. sphaeroides* was described by Erb *et al.* (1), and the plasmid pTE22 was used for production of the protein. The cloning and production of the helper enzymes ethylmalonyl-CoA mutase and epimerase were described by Erb *et al.* (28) and Schwander *et al.* (19).

Site-directed mutagenesis was performed with the Quik Change® method and protocols (Stratagene, La Jolla, CA). For the introduction of the mutations, 60 ng of template DNA and the corresponding primers were used (Table S1).

### Purification of recombinant enzymes

For the crystallization, the MCD from *P. denitrificans* was purified as follows. Cells were lysed by ultrasonification, and the lysate was cleared by ultracentrifugation at 50,000 rcf for 45 min at 4 °C and subsequently filtered through a 0.45-μm filter. The cleared lysate was loaded onto a 5-ml HisTrap FF column (GE Healthcare), and unbound protein was removed with 20 ml of 20 mm Tris-HCl, pH 7.9, 500 mm NaCl, 75 mm imidazole. The protein was eluted in 20 mm Tris-HCl, pH 7.9, 500 mm NaCl, and 500 mm imidazole and subsequently desalted with a HiTrap 5-ml desalting (GE Healthcare) column in 20 mm Tris-HCl, pH 7.9, 250 mm NaCl. The His tag was removed by thrombin (T4648, Sigma-Aldrich, Munich, Germany)-mediated proteolysis (1 mg of thrombin per 10 mg of PdMCD) for 2 h at room temperature. The digested protein was passed through a 1-ml HisTrap FF column (GE Healthcare), and the flow-through was collected. The digested protein was further purified via a HiLoad 16/600 Superdex 200 pg (GE Healthcare) size-exclusion column in 20 mm Tris-HCl, pH 7.9, 200 mm NaCl. Elution fractions were concentrated with Amicon Ultra-4 centrifugal filters (Merck Millipore, Darmstadt, Germany), and the protein concentration was determined on a Nanodrop 2000 spectrophotometer (Thermo Scientific, St. Leon-Rot, Germany). The purifications of the RsMCD, RsMCO, and the helper enzymes ethylmalonyl-CoA mutase and epimerase have been described previously (1, 19, 28).

### Crystallization and structure determination

Crystals were grown at 16 °C using the sitting-drop vapor diffusion method. The PdMCD protein was adjusted to a concentration of 20 mg ml^−1^ in a buffer containing 20 mm Tris-HCl, pH 7.9, 200 mm NaCl with a final concentration of 1.5 mm FAD and 3 mm mesaconyl-CoA. The protein solution was mixed in a 1:1 ratio with the crystallization buffer containing 30% PEG 5000 monomethyl ether mesylate, 100 mm Tris, pH 8, and 200 mm LiSO_4_. Crystals were soaked in 30% glycerol before being plunged into liquid nitrogen for freezing. X-ray diffraction data were collected at the European Synchrotron Radiation Facility (Grenoble, France) (beamline ID29). The data were processed with XDS (29) and the CCP4 software package (30). The structure was solved using molecular replacement using the Phaser-MR and AutoBuild programs of the Phenix software package (31). The structure of a putative acyl-CoA dehydrogenase (PDB code 4N5F; 26.5% sequence identity) served as a search model for the molecular replacement. Additional manual modeling and ligand fitting were performed using COOT (32). Because the FAD cofactor assumed a bend (butterfly-like) conformation for the isoalloxazine ring, the molecular restraints for the refinements had to be modified. The isoalloxazine ring was therefore separated into two planes along the N5/N10 axis of the central pyrazine ring. Additionally, the restraints for the bond angles over N5 and N10 were relaxed. Water-picking and further refinements were done by Phenix. The atomic coordinates (PDB code 6ES9) and structure factors have been deposited in the Protein Data Bank.

### Analytical size-exclusion chromatography

The PdMCD WT and variants (Q115A, T116A, and E117A) were initially purified as follows. Cells were lysed by ultrasonification, and the lysate was cleared by ultracentrifugation at 50,000 rcf for 45 min at 4 °C and subsequently filtered through a 0.45-μm filter. The cleared lysate was loaded onto a 1-ml HisTrap FF column (GE Healthcare), and unbound protein was removed with 20 ml of 20 mm Tris-HCl, pH 7.9, 500 mm NaCl, 75 mm imidazole. The protein was eluted in 20 mm Tris-HCl, pH 7.9, 500 mm NaCl, and 500 mm imidazole and subsequently desalted with a HiTrap 5-ml desalting (GE Healthcare) column in 20 mm Tris-HCl, pH 7.9, 200 mm NaCl. After the addition of 20% glycerol, the enzymes were concentrated to 10 mg ml^−1^ and incubated with 500 μm FAD for 1 h on ice. The enzymes were diluted to 2 mg ml^−1^ in 20 mm Tris-HCl, pH 7.9, 200 mm NaCl. 100 μl were injected onto a Superdex 200 Increase 10/300 GL size-exclusion column (GE Healthcare) with a flow rate of 0.7 ml min^−1^ and a running buffer of 20 mm Tris-HCl, pH 7.9, 200 mm NaCl. The size-exclusion calibration curve was obtained using the gel filtration standard (Bio-Rad).

### Spectrophotometric enzyme activity assay

The activity of PdMCD and RsMCD was determined using the protocol based on the reduction of ferrocenium as electron acceptor, which has been described before (1, 33). The reaction was followed at 300 nm (adjusted Δϵ_300_ = 2.75 mm^−1^ cm^−1^) on a Carry 60 UV-visible spectrophotometer (Agilent Technologies) at 30 °C. The reaction mixture (0.1 ml) contained 50 mm potassium phosphate buffer (pH 7.5), 0.25 mm ferrocenium hexafluorophosphate. For the assay with succinyl-CoA, the assay contained a 1.6 μm concentration of the RsMCD WT or A282V variant (which corresponds to Ala-285 in PdMCD). This assay was started by the addition of different concentrations of succinyl-CoA (10–800 μm). For the activity of the enzymes with (2*S*)-methylsuccinyl-CoA, the reaction contained additionally 7.5 μm ethylmalonyl-CoA mutase and 3 μm epimerase. The following enzyme concentrations were used in this case: 0.008 μm RsMCD WT, 0.01 μm RsMCD A282V (which corresponds to Ala-285 PdMCD), 1.9 μm RsMCD A282F/F284A (which corresponds to Ala-285/Phe-287 in PdMCD), or 0.007 μm PdMCD WT. The assay was started by the addition of varying substrate (ethylmalonyl-CoA) concentrations (10–1600 μm). To observe any influence of the artificial electron acceptor (ferrocenium), the same assay was conducted with a fixed substrate concentration (200 μm ethylmalonyl-CoA), and the ferrocenium concentration was varied between 100 and 250 μm.

### HPLC-MS–based assay of MCD

For the assay of the R252A/Q/K variants of PdMCD, single variants of RsMCD (A282V/L/I/F, which corresponds to Ala-285 in PdMCD) and the double variants of RsMCD (A282F with F284A/V/L, which corresponds to Ala-285 and Phe-287 in PdMCD), the reaction mixture (275 μl) contained 91 mm MOPS at pH 7.5 and 1.8 mm ferrocenium hexafluorophosphate. The assay with succinyl-CoA contained 34 μg of enzyme and was started with the addition of 680 μm succinyl-CoA. The assay with the natural substrate contained 1 μg of MCD variants, 110 μg of ethylmalonyl-CoA mutase, and 13 μg of epimerase. To start the reaction, 680 μm ethylmalonyl-CoA was added. Samples (30 μl) were withdrawn at certain time points, and the reaction was quenched in 3 μl of 40% formic acid.

To measure oxidase activity, the assay (100 μl) contained 100 μg of either RsMCD WT, RsMCD A282V, or RsMCO (19). Additionally, 7.5 μm ethylmalonyl-CoA mutase and 3 μm epimerase were added, and the assay was started with the addition of 200 μm ethylmalonyl-CoA. Samples (30 μl) were withdrawn at certain time points, and the reaction was quenched in 3 μl of 40% formic acid. All samples were analyzed by HPLC-MS as described below.

### HPLC-MS analysis

The CoA thioesters were analyzed using a 6550 iFunnel Q-TOF LC-MS system (Agilent Technologies) equipped with an electrospray ionization source set to positive ionization mode. Compounds were separated on an RP-18 column (50 × 2.1 mm, particle size 1.7 μm; Kinetex XB-C18, Phenomenex, Aschaffenburg, Germany) using a mobile phase system composed of 50 mm ammonium formate, pH 8.1, and methanol as described before (19). LC-MS data were analyzed using MassHunter qualitative analysis software (Agilent Technologies) and eMZed (34).

## Author contributions

T.S., R.M., J.Z., and T.J.E. conceptualization; T.S., R.M., J.Z., and T.J.E. formal analysis; T.S., R.M., J.Z., and T.J.E. investigation; T.S. and J.Z. visualization; T.S., R.M., J.Z., and T.J.E. writing-original draft; T.S., R.M., J.Z., and T.J.E. writing-review and editing; T.J.E. supervision; R.M. and T.J.E. funding acquisition.

## Supplementary Material

Supporting Information

## Footnotes

3 The abbreviations used are:

MCD: methylsuccinyl-CoA dehydrogenase
ACD: acyl-CoA dehydrogenase
ACO: acyl-CoA oxidase
ETF: electron transfer flavoprotein
GDH: glutaryl-CoA dehydrogenase
IVD: isovaleryl-CoA dehydrogenase
IBD: isobutyryl-CoA dehydrogenase
MCAD: medium-chain acyl-CoA dehydrogenase
PdMCD: methylsuccinyl-CoA dehydrogenase from *P. denitrificans*
RsMCD: methylsuccinyl-CoA dehydrogenase from *R. sphaeroides*
RsMCO: methylsuccinyl-CoA oxidase from *R. sphaeroides*
SCAD: short-chain acyl-CoA dehydrogenase
VLCAD: very-long-chain acyl-CoA dehydrogenase
PDB: Protein Data Bank
rcf: relative centrifugal force.
