# Supplementary material for: Structural basis for substrate specificity of methylsuccinyl-CoA dehydrogenase, an unusual member of the acyl-CoA dehydrogenase family
Source: J Biol Chem. 2017 Dec 22;293(5):1702–12. doi: 10.1074/jbc.RA117.000764 (PMC5798300; doi:10.1074/jbc.RA117.000764)
Supplement: Supporting Information [file supp_293_5_1702__index.html]

Structural basis for substrate specificity of methylsuccinyl-CoA dehydrogenase: an unusual member of the acyl-CoA dehydrogenase family. — Crystal structure of methylsuccinyl-CoA dehydrogenase — Structural basis for substrate specificity of methylsuccinyl-CoA dehydrogenase, an unusual member of the acyl-CoA dehydrogenase family — Crystal structure of methylsuccinyl-CoA dehydrogenase — Supporting Information 

# Structural basis for substrate specificity of methylsuccinyl-CoA dehydrogenase, an unusual member of the acyl-CoA dehydrogenase family

## Supporting Information

- Supplemental Data - Table S1 and Figures S1-S6
