# Supplementary material for: Structural basis for substrate specificity of methylsuccinyl-CoA dehydrogenase, an unusual member of the acyl-CoA dehydrogenase family
Source: J Biol Chem. 2017 Dec 22;293(5):1702–12. doi: 10.1074/jbc.RA117.000764 (PMC5798300; doi:10.1074/jbc.RA117.000764)
Supplement: Supporting Information [file supp_RA117.000764_133636_1_supp_41853_p19p30.pdf]

Supplemental Data

Structural basis for substrate specificity of methylsuccinyl-CoA dehydrogenase: an unusual member of the acyl-CoA dehydrogenase family.

Thomas Schwander[1], Richard McLean[1], Jan Zarzycki[1], and Tobias J. Erb[1,2]

[1] Max-Planck-Institute for Terrestrial Microbiology Marburg, Department of Biochemistry and Synthetic Metabolism, Karl-von-Frisch-Str. 10, D-35043 Marburg, Germany; [2] Center for Synthetic Microbiology, Philipps Universität Marburg, Germany.

Running title: *Crystal structure of methylsuccinyl-CoA dehydrogenase*

Table S1. Primers used for Site directed mutagenesis

Gene	Mutation	Fw Primer 5’→3’	Rv Primer 5’→3’	Template	Vector
**mcd R. sph.**	A282F	GATCCTGCCGACCTT CGTCTTCACCGAG	CTCGGTGAAGACGAA GGTCGGCAGGATC	pTE22	pTE835
**mcd R. sph.**	A282L	GATCCTGCCGACCCT CGTCTTCACCGAG	CTCGGTGAAGACGAG GGTCGGCAGGATC	pTE22	pTE836
**mcd R. sph.**	A282V	GATCCTGCCGACCGT CGTCTTCACCGAG	CTCGGTGAAGACGAC GGTCGGCAGGATC	pTE22	pTE837
**mcd R. sph.**	A282I	GATCCTGCCGACCAT CGTCTTCACCGAG	CTCGGTGAAGACGAT GGTCGGCAGGATC	pTE22	pTE843
**mcd R. sph**	A282F F284A	CCGACCTTCGTCGCC ACCGAGCCGAAC	GTTCGGCTCGGTGGC GACGAAGGTCGG	pTE835	pTE870
**mcd R. sph**	A282F F284V	GCCGACCTTCGTCGT CACCGAGCCGAAC	GTTCGGCTCGGTGAC GACGAAGGTCGGC	pTE835	pTE871
**mcd R. sph**	A282F F284L	GCCGACCTTCGTCCT CACCGAGCCGAAC	GTTCGGCTCGGTGAG GACGAAGGTCGGC	pTE835	pTE872
**mcd P. den.**	R252A	CTCGCTGGGCACCGC CAGCGAGATCGC	GCGATCTCGCTGGCG GTGCCCAGCGAG	pTE849	pTE860
**mcd P. den.**	R252K	CTCGCTGGGCACCAA AAGCGAGATCGCGG	CCGCGATCTCGCTTT TGGTGCCCAGCGAG	pTE849	pTE861
**mcd P. den.**	R252Q	CGCTGGGCACCCAGA GCGAGATCGC	GCGATCTCGCTCTGG GTGCCCAGCG	pTE849	pTE862
**mcd P. den.**	Q115A	CATCCCGATGAGCGC GACGGAGTTCGC	GCGAACTCCGTCGCG CTCATCGGGATG	pTE849	pTE873
**mcd P. den.**	T116A	CCGATGAGCCAGGCG GAGTTCGCCC	GGGCGAACTCCGCCT GGCTCATCGG	pTE849	pTE874
**mcd P. den.**	E117A	GATGAGCCAGACGGC GTTCGCCCGCCTC	GAGGCGGGCGAACG CCGTCTGGCTCATC	pTE849	pTE875

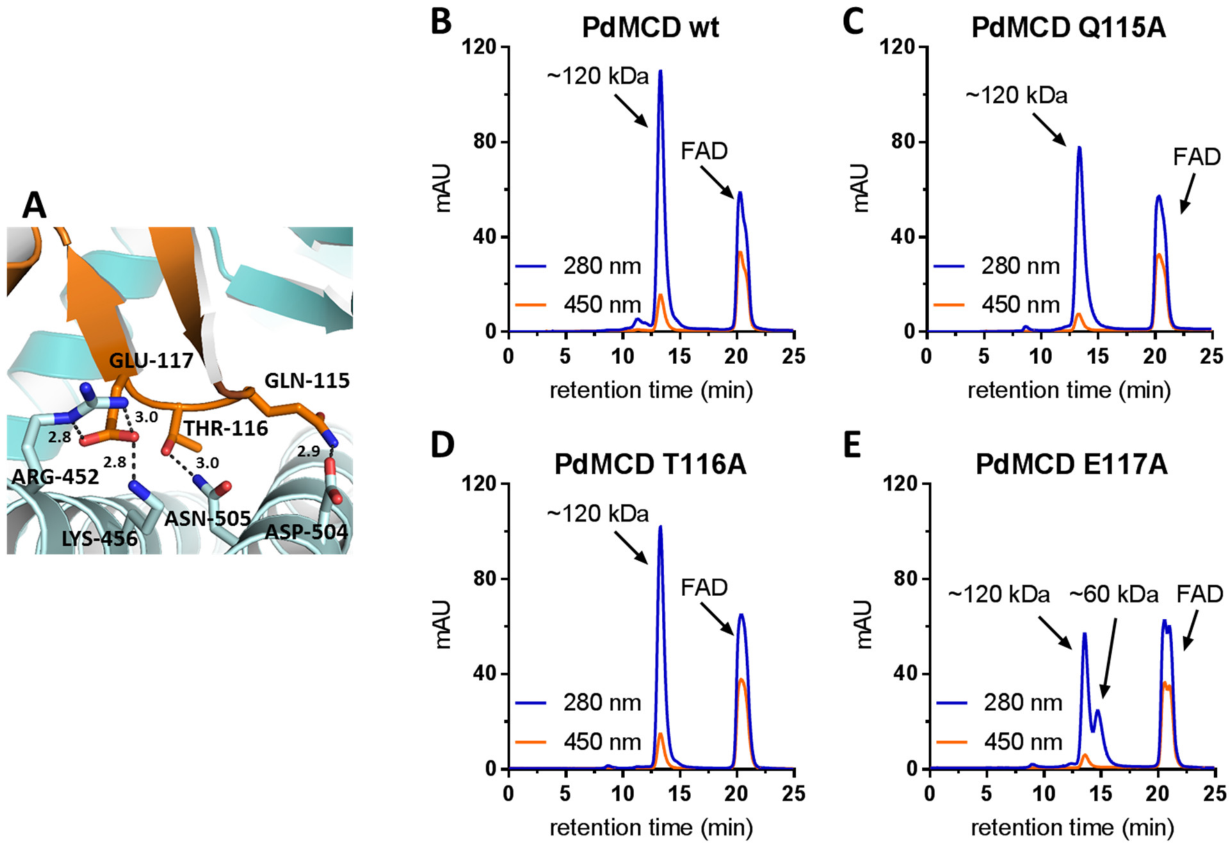

Figure S1. (A) The β-hairpin motif comprises three residues (Q115, T116 and E117) in the connecting loop, which interact with the central domain of the neighboring subunit. (B-D) Analytical size exclusion chromatogram of the PdMCD wt and two alanine variants (Q115A and T116A) indicating a dimeric state (~120 kDa), which were able to bind FAD (detected at 450 nm). Note, that the enzymes were pre-incubated with 500 µM of FAD resulting in the observed FAD peak after 20 min. (E) Analytical size exclusion chromatogram of the PdMCD E117A variant, which was impaired in dimer formation leading to a corresponding monomer peak of ~60 kDa. In the monomeric form bound FAD was not observable, because both subunits contribute to the binding of the FAD cofactor.

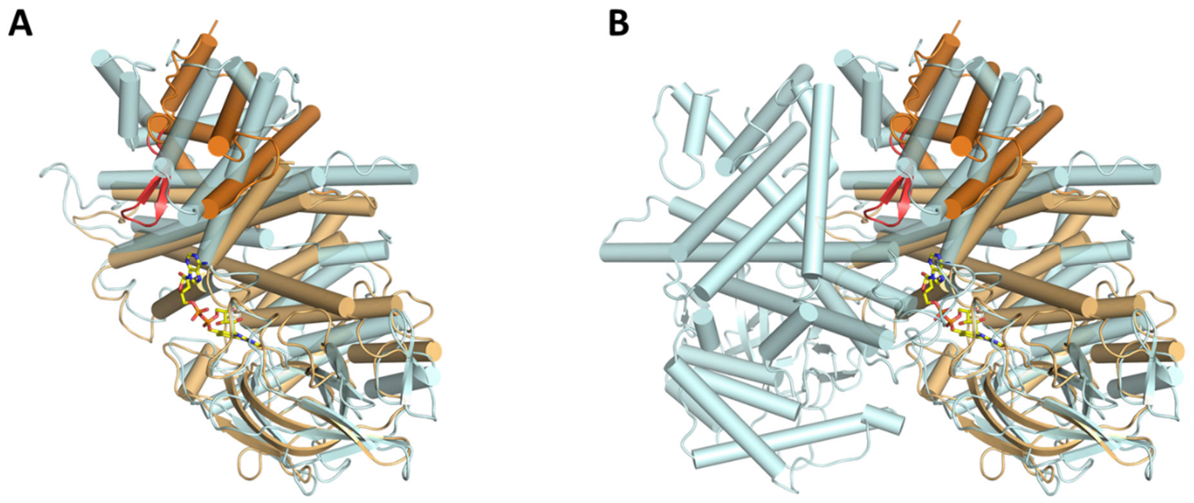

Figure S2. (A) Superposition of a MCD monomer in orange (N-terminal domain in dark orange) with an ACO monomer (PDB ID: 1IS2) (4.82 Å over 591 Cα-atoms). The central fold is also conserved in ACOs and the structures aligns to some extent. (B) Overlay of a MCD monomer with the dimer of ACO in cyan.

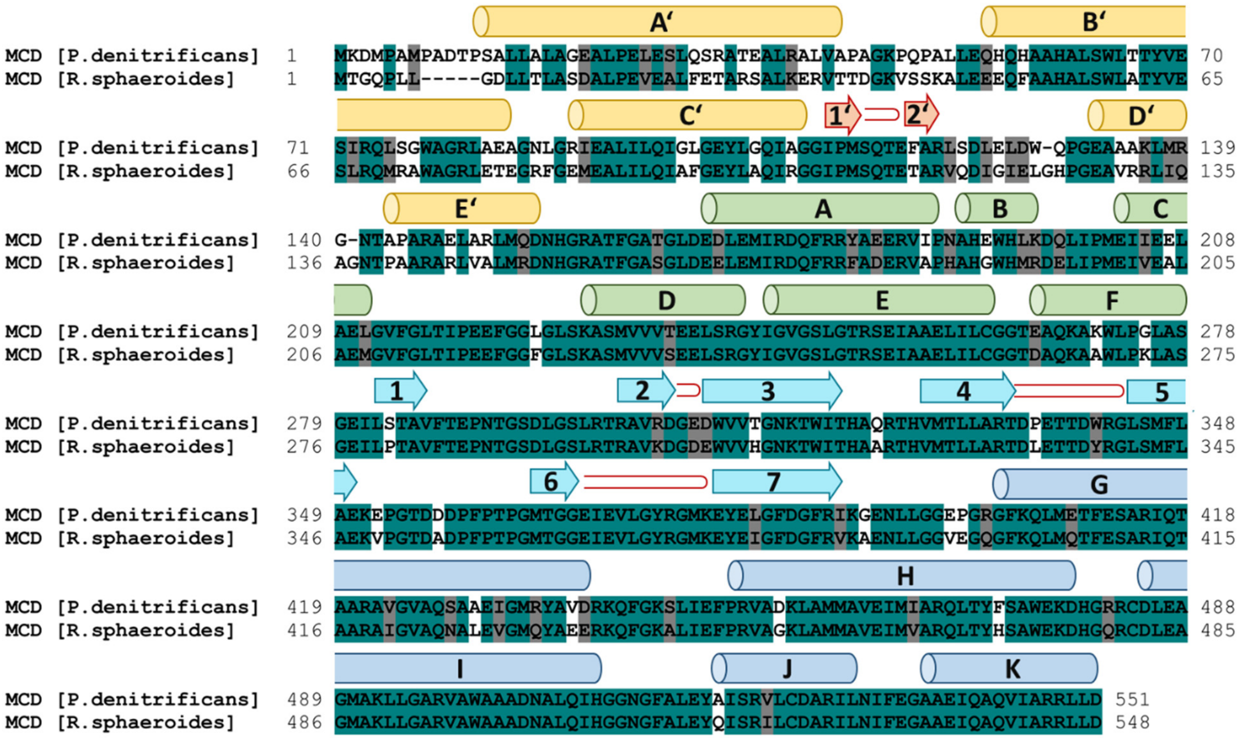

Figure S3. The sequence alignment of the MCDs of *Paracoccus denitrificans* and *Rhodobacter sphaeroides* shows an overall identity of 77.8% and a similarity of 86.1%. The catalytic core domain (without the N-terminal extension) shows 88% identity and 95% similarity in the amino acid sequence.

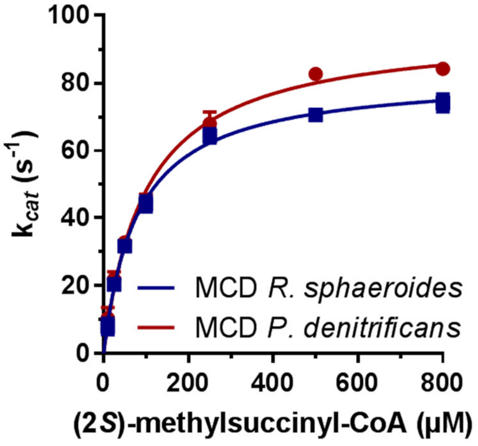

	(2*S*)-methylsuccinyl-CoA k_{cat} (s^{-1})	app. K_M (µM)	k_{cat}/K_M (s^{-1} M^{-1})
**MCD R.sphaeroides**	82.3 ± 1.4	80 ± 5	$1.0 \times 10^6 \pm 0.1 \times 10^6$
**MCD P.denitrificans**	95.9 ± 2.2	99 ± 8	$1.0 \times 10^6 \pm 0.1 \times 10^6$

Figure S4. Michaelis-Menten kinetics of the MCDs of *Rhodobacter sphaeroides* and *Paracoccus denitrificans* with (2*S*)-methylsuccinyl-CoA as substrate. Both enzymes show very similar catalytic properties.

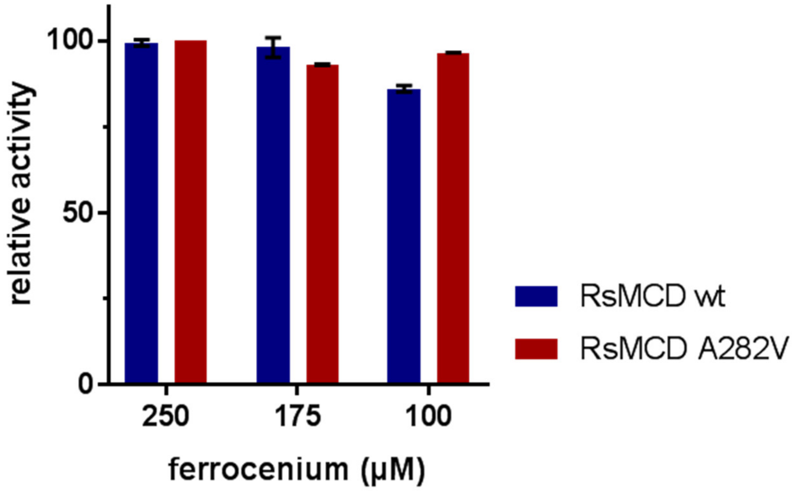

Figure S5. Relative activities of RsMCD wt and A282V variant with 200 µM of (2*S*)-methylsuccinyl-CoA and different concentrations of ferrocenium. The activity of both enzymes were not dependent on the concentration of ferrocenium under these conditions.

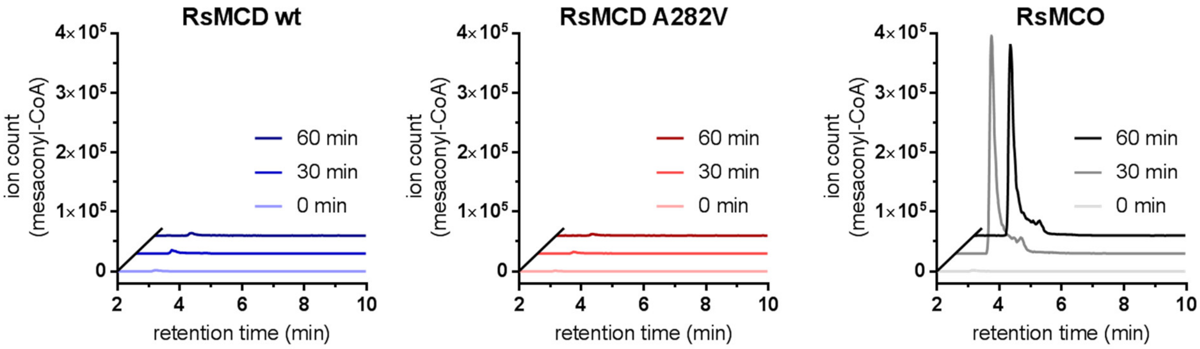

Figure S6. HPLC-MS analysis of the oxidase activities for the A282V variant with (2*S*)-methylsuccinyl-CoA as substrate and molecular dioxygen (air saturated solutions) as electron acceptor. The introduced A282V mutation did not affect the oxidase activity of the variant. Note, that the control is an oxidase active variant of RsMCD (RsMCO).
